# Post–COVID Conditions Among Adult COVID-19 Survivors Aged 18–64 and ≥65 Years — United States, March 2020–November 2021

**DOI:** 10.15585/mmwr.mm7121e1

**Published:** 2022-05-27

**Authors:** Lara Bull-Otterson, Sarah Baca, Sharon Saydah, Tegan K. Boehmer, Stacey Adjei, Simone Gray, Aaron M. Harris

**Affiliations:** ^1^CDC COVID-19 Emergency Response Team; ^2^GAP Solutions, Inc., Herndon, Virginia.

A growing number of persons previously infected with SARS-CoV-2, the virus that causes COVID-19, have reported persistent symptoms, or the onset of long-term symptoms, ≥4 weeks after acute COVID-19; these symptoms are commonly referred to as post-COVID conditions, or long COVID (*1*). Electronic health record (EHR) data during March 2020–November 2021, for persons in the United States aged ≥18 years were used to assess the incidence of 26 conditions often attributable to post-COVID (hereafter also referred to as incident conditions) among patients who had received a previous COVID-19 diagnosis (case-patients) compared with the incidence among matched patients without evidence of COVID-19 in the EHR (control patients). The analysis was stratified by two age groups (persons aged 18–64 and ≥65 years). Patients were followed for 30–365 days after the index encounter until one or more incident conditions were observed or through October 31, 2021 (whichever occurred first). Among all patients aged ≥18 years, 38% of case-patients experienced an incident condition compared with 16% of controls; conditions affected multiple systems, and included cardiovascular, pulmonary, hematologic, renal, endocrine, gastrointestinal, musculoskeletal, neurologic, and psychiatric signs and symptoms. By age group, the highest risk ratios (RRs) were for acute pulmonary embolism (RR = 2.1 and 2.2 among persons aged 18–64 and ≥65 years, respectively) and respiratory signs and symptoms (RR = 2.1 in both age groups). Among those aged 18–64 years, 35.4% of case-patients experienced an incident condition compared with 14.6% of controls. Among those aged ≥65 years, 45.4% of case-patients experienced an incident condition compared with 18.5% of controls. These findings translate to one in five COVID-19 survivors aged 18–64 years, and one in four survivors aged ≥65 years experiencing an incident condition that might be attributable to previous COVID-19. Implementation of COVID-19 prevention strategies, as well as routine assessment for post-COVID conditions among persons who survive COVID-19, is critical to reducing the incidence and impact of post-COVID, particularly among adults aged ≥65 years (*2*).

A retrospective matched cohort design was used to analyze EHRs during March 2020–November 2021, from Cerner Real-World Data,* a national, deidentified data set of approximately 63.4 million unique adult records from 110 data contributors in the 50 states. Case-patients (353,164) were adults aged ≥18 years who received either a diagnosis of COVID-19 or a positive SARS-CoV-2 test result^†^ (case-patient index encounter) in an inpatient, emergency department, or outpatient settings within a subset of health care facilities that use Cerner EHRs. Control patients (1,640,776) had a visit in the same month as the matched case-patient (control index encounter) and did not receive a COVID-19 diagnosis or a positive SARS-CoV-2 test result during the observation period. Controls were matched 5:1 with case-patients. All patients included in the analysis were required to have at least one encounter in their EHR during the year preceding and the year after the index encounter.

The occurrence of 26 clinical conditions previously attributed to post-COVID illness was assessed by review of the scientific literature^§^ (*3*–*5*) (Supplementary Table 1, https://stacks.cdc.gov/view/cdc/117411). Patients were followed for 30–365 days after the index encounter until the first occurrence of an incident condition or until October 31, 2021, whichever occurred first. Case-patients or control patients with a previous history of one of the included conditions in the year before the index encounter were excluded (478,072 patients). The analysis was stratified by age into two groups: adults aged 18–64 and adults aged ≥65 years. Incidence rates per 100 person-months, and RRs with 95% CIs, were calculated. The number of COVID-19 case-patients having experienced an incident condition was also estimated by age group.^¶^ Nonoverlapping CIs between age groups were considered statistically significant. Analyses were performed using RStudio Workbench (version 3.0; RStudio). This activity was reviewed by CDC and was conducted consistent with applicable federal law and CDC policy.**

Among all patients aged ≥18 years, 38.2% of case-patients and 16.0% of controls experienced at least one incident condition (Table). Among persons aged 18–64 years, 35.4% of case-patients and 14.6% of controls experienced at least one incident condition. Among persons aged ≥65 years, 45.4% of case-patients and 18.5% of controls experienced at least one incident condition. The absolute risk difference between the percentage of case-patients and controls who developed an incident condition was 20.8 percentage points for those aged 18–64 years and 26.9 percentage points for those aged ≥65 years. This finding translates to one in five COVID-19 survivors aged 18–64 years and one in four survivors aged ≥65 years experiencing an incident condition that might be attributable to previous COVID-19.

**TABLE:** Percentage of adult COVID-19 case-patients and control patients with ≥1 post-COVID–attributable incident conditions and estimated number of COVID-19 survivors who will experience a post-COVID condition — United States, March 2020–November 2021

Age group, yrs	No. of patients (column %)		No. of patients with ≥1 incident condition (column %*)		Absolute risk difference^†^	No. of COVID-19 survivors with a post-COVID condition^§^
	Case-patients	Control patients	Case-patients	Control patients		
18–64	254,345 (72.0)	1,051,588 (64.1)	90,111 (35.4)	154,011 (14.6)	20.8	1/5
≥65	98,819 (28.0)	589,188 (35.9)	44,840 (45.4)	108,850 (18.5)	26.9	1/4
**Total**	**353,164 (100)**	**1,640,776 (100)**	**134,951 (38.2)**	**262,861 (16.0)**	**22.2**	**1/4–5**

* Percentage of COVID-19 case-patients or control patients with ≥1 incident condition divided by the total study COVID-19 cohort or control cohort row’s age group total.

^†^ Percentage point difference between COVID-19 case-patients and control patients (e.g., the value 20.8 is calculated as 35.4 minus 14.6).

^§^ Number of COVID-19 survivors who experienced a post-COVID condition estimated as the inverse of the absolute risk difference.

The most common incident conditions in both age groups were respiratory symptoms and musculoskeletal pain (Supplementary Table 2, https://stacks.cdc.gov/view/cdc/117411). Among both age groups, the highest RRs were for incident conditions involving the pulmonary system, including acute pulmonary embolism (RR = 2.2 [patients aged ≥65 years] and 2.1 [patients aged 18–64 years]) and respiratory symptoms (RR = 2.1, both age groups) (Figure). Among patients aged ≥65 years, the risks were higher among case-patients than among controls for all 26 incident conditions, with RRs ranging from 1.2 (substance-related disorder) to 2.2 (acute pulmonary embolism). Among patients aged 18–64 years, the risks were higher among case-patients than among controls for 22 incident conditions, with RRs ranging from 1.1 (anxiety) to 2.1 (acute pulmonary embolism); no significant difference was observed for cerebrovascular disease, or mental health conditions, such as mood disorders, other mental conditions, and substance-related disorders.

**FIGURE:**
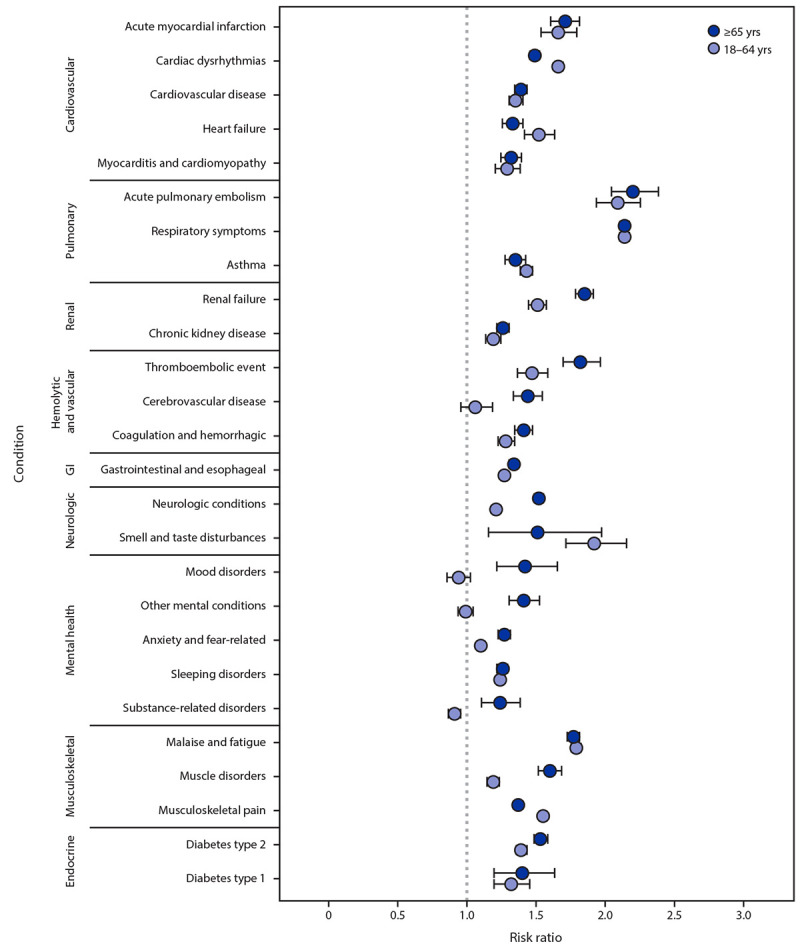
Risk ratios* for developing post-COVID conditions among adults aged 18–64 years and ≥65 years — United States, March 2020– November 2021 **Abbreviation: **GI = gastrointestinal. * With CIs indicated by error bars; some error bars are not visible because of small CIs.

Differences by age group were noted. The RR for cardiac dysrhythmia was significantly higher among patients aged 18–64 years (RR = 1.7) compared with those aged ≥65 years (1.5). Similarly, the RR for musculoskeletal pain was higher among patients aged 18–64 years (1.6) than among those aged ≥65 years (1.4). Among case-patients, the RRs for 10 incident conditions was significantly higher among those aged ≥65 years than among those aged 18–64 years; these conditions were renal failure, thromboembolic events, cerebrovascular disease, type 2 diabetes, muscle disorders, neurologic conditions, and mental health conditions (including mood disorders, anxiety, other mental conditions, and substance-related disorders).

## Discussion

The findings from this analysis of a large EHR-based database of U.S. adults indicated that COVID-19 survivors were significantly more likely than were control patients to have incident conditions that might be attributable to previous COVID-19. One in five COVID-19 survivors aged 18–64 years and one in four survivors aged ≥65 years experienced at least one incident condition that might be attributable to previous COVID-19. Independent of age group, the highest RRs were for acute pulmonary embolism and respiratory symptoms.

These findings are consistent with those from several large studies that indicated that post-COVID incident conditions occur in 20%–30% of patients (*6*,*7*), and that a proportion of patients require expanded follow-up care after the initial infection. COVID-19 severity and illness duration can affect patients’ health care needs and economic well-being (*8*). The occurrence of incident conditions following infection might also affect a patient’s ability to contribute to the workforce and might have economic consequences for survivors and their dependents, particularly among adults aged 18–64 years (*5*). In addition, care requirements might place a strain on health services after acute illness in communities that experience heavy COVID-19 case surges.

COVID-19 survivors aged ≥65 years in this study were at increased risk for neurologic conditions, as well as for four of five mental health conditions (mood disorders, other mental conditions, anxiety, and substance-related disorders). Neurocognitive symptoms have been reported to persist for up to 1 year after acute infection and might persist longer (*9*). Overall, 45.4% of survivors aged ≥65 years in this study had incident conditions. Among adults aged ≥65 years, who are already at higher risk for stroke and neurocognitive impairment, post-COVID conditions affecting the nervous system are of particular concern because these conditions can lead to early entry into supportive services or investment of additional resources into care (*10*).

The findings in this study are subject to at least five limitations. First, patient data were limited to those seen at facilities serviced by Cerner EHR network during January 2020–November 2021; therefore, the findings might not be representative of the entire U.S. adult population or of COVID-19 case patients infected with recent variants. Second, the incidence of new conditions after an acute COVID-19 infection might be biased toward a population that is seeking care, either as a follow-up to a previous complaint (including COVID-19) or for another medical complaint, which might result in a “sicker” control group leading to underestimation of observed risk. Third, COVID-19 vaccination status was not considered in this analysis, nor were potentially confounding factors (e.g., SARS-CoV-2 variant, sex, race, ethnicity, health care entity, or geographic region), because data were not available, were inconsistent, or included a high proportion of missing or unknown values; for example, data were not matched by data contributors, so controls were not necessarily from the same health care entity or region of the country. Differences between the groups might influence the risks associated with survival from COVID-19 and incident conditions, which require further study. Fourth, *International Classification of Disease, Tenth Revision, Clinical Modification* (ICD-10-CM) codes were used to identify COVID-19 case-patients, and misclassification of controls is possible. However, the inclusion of laboratory data to identify case-patients and exclude controls helped to limit the potential for such misclassification. Finally, the study only assessed conditions thought to be attributable to COVID-19 or post-COVID illness, which might have biased RRs away from the null. For example, clinicians might have been more likely to document possible post-COVID conditions among case-patients. In addition, because several conditions examined are also risk factors for moderate to severe COVID-19, it is possible that case-patients were more likely to have had an existing condition that was not documented in their EHR during the year preceding their COVID-19 diagnosis, resulting in overestimated risk for this group.

As the cumulative number of persons ever having been infected with SARS-CoV-2 increases, the number of survivors suffering post-COVID conditions is also likely to increase. Therefore, implementation of COVID-19 prevention strategies, as well as routine assessment for post-COVID conditions among persons who survive COVID-19, is critical to reducing the incidence and impact of post-COVID conditions, particularly among adults aged ≥65 years (*2*). These findings can increase awareness for post-COVID conditions and improve post-acute care and management of patients after illness. Further investigation is warranted to understand the pathophysiologic mechanisms associated with increased risk for post-COVID conditions, including by age and type of condition.

**Summary**

**What is already known about this topic?**

As more persons are exposed to and infected by SARS-CoV-2, reports of patients who experience persistent symptoms or organ dysfunction after acute COVID-19 and develop post-COVID conditions have increased.

**What is added by this report?**

COVID-19 survivors have twice the risk for developing pulmonary embolism or respiratory conditions; one in five COVID-19 survivors aged 18–64 years and one in four survivors aged ≥65 years experienced at least one incident condition that might be attributable to previous COVID-19.

**What are the implications for public health practice?**

Implementation of COVID-19 prevention strategies, as well as routine assessment for post-COVID conditions among persons who survive COVID-19, is critical to reducing the incidence and impact of post-COVID conditions, particularly among adults aged ≥65 years.

## Footnotes

*
https://www.cerner.com/solutions/real-world-data?_ga=2.134259058.2081252678.1649198012-1806687702.1649105445

† COVID-19 cases with associated positive test result were identified by the following: Systematized Nomenclature of Medicine (SNOMED) codes 840533007, 840535000, 840539006, and 840546002; *International Classification of Diseases, Tenth Edition, Clinical Modification* (ICD-10-CM) codes B97.29 (March, 2020) and U07.1 (April–May 2020); and Logical Observation Identifiers Names and Codes (LOINC) codes 68993–5, 92142–9, 92141–1, 94309–2, 94307–6, 94308–4, 94500–6, 94502–2, 94533–7, 94534–5, 94559–2, 94756–4, 94757–2, 94758–0, 94845–5, 95406–5, 95409–9, 96091–4, 95425–5, 95423–0, and 96448–6.

§ Acute myocardial infarction, cardiac dysrhythmias, cardiovascular disease, heart failure, myocarditis and cardiomyopathy, acute pulmonary embolism, respiratory symptoms, asthma, renal failure, chronic kidney disease, thromboembolic event, cerebrovascular disease, coagulation and hemorrhagic conditions, gastrointestinal and esophageal conditions, neurologic conditions, smell and taste disturbances, mood disorders, other mental conditions, anxiety and fear-related conditions, sleeping disorders, substance-related disorders, malaise and fatigue, muscle disorders, musculoskeletal pain, diabetes type 2, and diabetes type 1.

¶ Calculated as the reciprocal of the absolute risk difference of COVID-19 case-patients and non–COVID-19 controls that experience at least one incident condition.

** 45 C.F.R. part 46.102(l)(2), 21 C.F.R. part 56; 42 U.S.C. Sect. 241(d); 5 U.S.C. Sect. 552a; 44 U.S.C. Sect. 3501 et seq

## References

[1] CDC. Long COVID or post-COVID conditions. Atlanta, GA: US Department of Health and Human Services, CDC; 2022. Accessed April 22, 2022. https://www.cdc.gov/coronavirus/2019-ncov/long-term-effects/index.html

[2] Antonelli M, Penfold RS, Merino J, Risk factors and disease profile of post-vaccination SARS-CoV-2 infection in UK users of the COVID Symptom Study app: a prospective, community-based, nested, case-control study. Lancet Infect Dis 2022;22:43-55. 10.1016/S1473-3099(21)00460-6 34480857 PMC8409907

[3] Al-Aly Z, Xie Y, Bowe B. High-dimensional characterization of post-acute sequelae of COVID-19. Nature 2021;594:259–64. 10.1093/cid/ciab338 33887749

[4] Cohen K, Ren S, Heath K, Risk of persistent and new clinical sequelae among adults aged 65 years and older during the post-acute phase of SARS-CoV-2 infection: retrospective cohort study. BMJ 2022;376:e068414. 10.1136/bmj-2021-068414 35140117 PMC8828141

[5] Rajan S, Khunti K, Alwan N, In the wake of the pandemic: preparing for long COVID. Copenhagen, Denmark: European Observatory on Health Systems and Policies; 2021. 33877759

[6] Ayoubkhani D, Khunti K, Nafilyan V, Post-COVID syndrome in individuals admitted to hospital with COVID-19: retrospective cohort study. BMJ 2021;372:n693. 10.1136/bmj.n693 33789877 PMC8010267

[7] Donnelly JP, Wang XQ, Iwashyna TJ, Prescott HC. Readmission and death after initial hospital discharge among patients with COVID-19 in a large multihospital system. JAMA 2021;325:304–6. 10.1001/jama.2020.21465 33315057 PMC7737131

[8] CDC. Science brief: indicators for monitoring COVID-19 community levels and making public health recommendations. Atlanta, GA: US Department of Health and Human Services; 2022. https://www.cdc.gov/coronavirus/2019-ncov/science/science-briefs/indicators-monitoring-community-levels.html 35324136

[9] Mueller AL, McNamara MS, Sinclair DA. Why does COVID-19 disproportionately affect older people? Aging (Albany NY) 2020;12:9959–81. 10.18632/aging.103344 32470948 PMC7288963

[10] Mohamed MS, Johansson A, Jonsson J, Schiöth HB. Dissecting the molecular mechanisms surrounding post-COVID-19 syndrome and neurological features. Int J Mol Sci 2022;23:4275. 10.3390/ijms23084275 35457093 PMC9028501

